# Monoassociation of Preterm Germ-Free Piglets with *Bifidobacterium animalis* Subsp. *lactis* BB-12 and Its Impact on Infection with *Salmonella* Typhimurium

**DOI:** 10.3390/biomedicines9020183

**Published:** 2021-02-11

**Authors:** Alla Splichalova, Sharon M. Donovan, Helena Tlaskalova-Hogenova, Zbynek Stranak, Zdislava Splichalova, Igor Splichal

**Affiliations:** 1 Laboratory of Gnotobiology, Institute of Microbiology, Czech Academy of Sciences, 549 22 Novy Hradek, Czech Republic; splichalova@gnotobio.cz (A.S.); zdispl@gnotobio.cz (Z.S.); 2 Department of Food Science and Human Nutrition, University of Illinois, Urbana, IL 61801, USA; sdonovan@illinois.edu; 3 Laboratory of Cellular and Molecular Immunology, Institute of Microbiology, Czech Academy of Sciences, 142 20 Prague, Czech Republic; tlaskalo@biomed.cas.cz; 4 Department of Neonatology, Institute for the Care of Mother and Child, 147 00 Prague, Czech Republic; zbynek.stranak@upmd.eu

**Keywords:** *Bifidobacterium animalis* subsp. *lactis* BB-12, *Salmonella* Typhimurium, intestinal barrier, inflammatory cytokines, preterm host, immunocompromised

## Abstract

Preterm germ-free piglets were monoassociated with probiotic *Bifidobacterium animalis* subsp. *lactis* BB-12 (BB12) to verify its safety and to investigate possible protection against subsequent infection with *Salmonella* Typhimurium strain LT2 (LT2). Clinical signs of salmonellosis, bacterial colonization in the intestine, bacterial translocation to mesenteric lymph nodes (MLN), blood, liver, spleen, and lungs, histopathological changes in the ileum, claudin-1 and occludin mRNA expression in the ileum and colon, intestinal and plasma concentrations of IL-8, TNF-α, and IL-10 were evaluated. Both BB12 and LT2 colonized the intestine of the monoassociated piglets. BB12 did not translocate in the BB12-monoassociated piglets. BB12 was detected in some cases in the MLN of piglets, consequently infected with LT2, but reduced LT2 counts in the ileum and liver of these piglets. LT2 damaged the luminal structure of the ileum, but a previous association with BB12 mildly alleviated these changes. LT2 infection upregulated claudin-1 mRNA in the ileum and colon and downregulated occludin mRNA in the colon. Infection with LT2 increased levels of IL-8, TNF-α, and IL-10 in the intestine and plasma, and BB12 mildly downregulated them compared to LT2 alone. Despite reductions in bacterial translocation and inflammatory cytokines, clinical signs of LT2 infection were not significantly affected by the probiotic BB12. Thus, we hypothesize that multistrain bacterial colonization of preterm gnotobiotic piglets may be needed to enhance the protective effect against the infection with *S.* Typhimurium LT2.

## 1. Introduction

Mode of delivery, nutrition, and exposure to antibiotics are the most important factors influencing the microbial seeding of the gastrointestinal tract (GIT) and postnatal development of GIT microbial composition [1]. The initial colonizers in vaginally born babies are facultative anaerobes from the mother’s vaginal and intestinal microbiota. They create conditions for subsequent onset of strictly anaerobic species, e.g., *Bacteroides*, *Bifidobacterium*, and *Clostridium* spp. [2]. Breastfeeding contributes to the GIT microbiota development by providing other microbes, human milk oligosaccharides, and bioactive proteins [1]. Consequently, the microbiome of breastfed infants show higher Proteobacteria but lower Bacteroidetes and Firmicutes than formula-fed infants [3]. In contrast, the microbes from the skin and hospital environment are the cesarean section-born infants’ primary colonizers. These children have lower counts of bifidobacteria, but the occurrence of *Clostridium difficile* is more frequent [4,5].

Animal models of human diseases represent a useful tool to elucidate underlying pathogenetic mechanisms [6,7]. The pig shares similar anatomy and physiology to the human and is a suitable animal model for translational research, e.g., for gastrointestinal diseases [8], infections [9], and sepsis [10]. The composition of the pig microbiome [11] is more similar to the human microbiome [12] than to the highly dissimilar mouse microbiome [13]. Thus, the pig is an excellent translational model to study host–microbiota interactions. In addition, the ability to colonize the pig GIT by human fecal microbiota was shown in conventional piglets [14] and later verified in experiments with germ-free (GF) piglets [15,16]. The GF animals are advantageous compared to their conventional counterparts because, if associated with the human microbiota, they enable investigations into host–microbiota interactions without an undefined background of the indigenous microbiota [17,18]. Lastly, the pig’s epitheliochorial placenta prevents the prenatal transfer of immunoglobulins and cells of maternal origin [19,20]; thus, cesarean section-derived, colostrum-deprived, GF piglets are immunocompromised, making them even a more similar model for the preterm infant [21].

Bifidobacteria are the most abundant intestinal bacteria in vaginally born and breast milk-fed infants [22,23]. They are obligate anaerobes that utilize host-indigestible complex carbohydrates derived from the host (e.g., mucin) or from the diet (e.g., human milk oligosaccharides or prebiotics) through specialized fermentative metabolic pathways [24]. Thus, they confer their beneficial effect by their saccharolytic activity on glycans that are abundant in the infant’s intestine [25] and produce growth substrates for other members of the gut microbial community [26]. Their impact on the host’s health is generally considered beneficial [22], and they are used for probiotic therapy in infants, including preterm ones [27]. However, there have case reports of bacteremia reported in preterm infants with probiotic preparations containing bifidobacteria [28,29].

Basic mechanisms of microbiota actions against enteric pathogens are known [30]. *Salmonella enterica* serovars represent such pathogens [31]. The serovar Typhimurium belongs to non-typhoidal *Salmonella* and causes self-limiting enterocolitis [32] in humans [33] and pigs [34], but typhoid fever-like disease in mice [35]. In the case of immunocompromised hosts, it can cause life-threatening extra-intestinal focal infections, including meningitis, osteomyelitis, septic arthritis, deep soft tissue infection, and pneumonia [36]. Increased bacterial multiresistance to antibiotics forces to find alternative ways to support the resistance of the immunocompromised host to infections [37,38]. One such alternative is using probiotics [39]. This work aimed to verify the safety of *Bifidobacterium animalis* subsp. *lactis* BB-12 for a preterm gnotobiotic piglet as a model of preterm infants and evaluate its ability to reduce the harmful effect of the infection with enteric pathogen *Salmonella* Typhimurium strain LT2.

## 2. Material and Methods

### 2.1. Bacterial Cultures and Inocula

*Bifidobacterium animalis* subsp. *lactis* BB-12 (BB12) was isolated from a commercial preparation Biopron Respiron (Valosun, Trinec, Czechia) containing BB12 only. The method of isolation was slightly modified from the method used for the isolation of *Bifidobacterium boum* [40]. Briefly, we resuspended BB12-containing powder in 0.05 M phosphate buffer, pH 6.5 containing 500 mg/L cysteine (PBC) and inoculated it onto Wilkins–Chalgren agar (Oxoid, Basingstoke, UK) supplemented with soya peptone (5 g/L; Oxoid), mupirocin (100 mg/L), and acetic acid (1 mL/L) in anaerobic jars with AnaeroGen sachets (Oxoid) and incubated at 37 °C for 48 h. After verifying by Gram-staining, the individual colonies were moved to vials containing 10 mL of Wilkins–Chalgren broth (Oxoid) supplemented with soya peptone (5 g/L, Oxoid) and anaerobically cultivated at 37 °C overnight. The cells were harvested by centrifugation at 4000× *g* for 10 min at RT, and the cell pellet was washed twice with 0.05 M phosphate buffer, pH 6.5 containing 500 mg/L cysteine (PBC).

*Salmonella enterica* serovar Typhimurium strain LT2 (*S*. Typhimurium or LT2) was from a collection of the microorganism of the Institute of Microbiology of the Czech Academy of Sciences (Novy Hradek, Czechia). *S*. Typhimurium was cultivated on meat-peptone agar slopes (blood agar base; Oxoid) at 37 °C overnight and after it scraped from the agar for preparation of the inoculum.

BB12 and LT2 cells were resuspended to 8 log CFU/mL density in PBC for oral administration to the piglets. The evaluated BB12 and LT2 cell densities at 600 nm were verified by cultivation methods on Wilkins–Chalgren (Oxoid) and MacConkey (Merck, Darmstadt, Germany) agars, respectively.

### 2.2. Preterm Gnotobiotic Piglets

**Figure 1:**
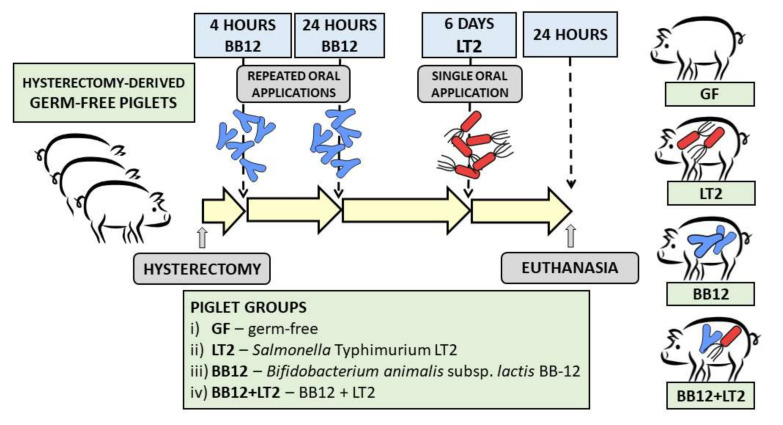
Experiment design. Gnotobiotic piglets (*n* = 24) were assigned into four groups with six piglets per group: (i) germ-free (GF); (ii) infected with *Salmonella* Typhimurium strain LT2 (LT2); (iii) associated with probiotic *Bifidobacterium animalis* subsp. *lactis* BB-12 (BB12); (iv) associated with BB12 and infected with LT2 (BB12 + LT2).

Preterm GF piglets were obtained by hysterectomy of pregnant miniature sows (Animal Research Institute, Kostelec nad Orlici, Czechia) on the 104th day of gestation. They were reared in fiberglass isolators with a heated floor, fed to satiety 6–7 times per day with cow’s milk-based formula by a nipple, and microbiologically tested as described in detail elsewhere [21]. A total number of 24 piglets were assigned to four groups with six piglets per group (Figure 1): (i) GF for the whole experiment, (ii) one-week-old GF piglets orally infected with 8 log CFU of LT2 for 24 h (LT2), (iii) orally associated with 8 log CFU of BB12 4 h and 24 h after hysterectomy (BB12); (iv) one-week-old BB12 piglets orally infected with 8 log CFU of LT2 for 24 h (BB12 + LT2). The piglets of each group were obtained from three independent hysterectomies. The bacterial inocula BB12 and LT2 were administered orally in 5 mL of the milk diet. The GF piglets received 5 mL of milk only. At the end of the experiment, the piglets were euthanized by exsanguination via cardiac puncture under isoflurane anesthesia.

### 2.3. Clinical Signs of Salmonellosis

The piglets of all groups were examined for fever, anorexia, sleepiness, and diarrhea every 3–4 h at the time of feeding.

### 2.4. Bacterial Counts in the Intestine and Translocation to Organs

The jejunum (40 cm segment of the proximal part of the jejunum) and ileum (40 cm segment of a distal part of the small intestine containing the distal jejunum and the ileum) were filled with 2 mL of PBC, gently kneaded, and rinsed. The entire colon was cut into small pieces on a 90 mm Petri dish and lavaged in 4 mL of PBC. Pieces of mesenteric lymph nodes, liver, and spleen—1 part (g) of the tissue and four parts (mL) deionized water were homogenized in a 2 mL Eppendorf tube with 3.2 mm stainless steel beads in a TissueLyser LT BeadBeater (Qiagen, Hilden, Germany). The lavages, tissue homogenates, and blood were log 10 serially diluted in PBC. The cultivation of BB12 was performed in 50 mm Petri dishes with Wilkins–Chalgren agar (Oxoid) supplemented with soya peptone (5 g/L, Oxoid), L-cysteine (0.5 g/L, Merck), mupirocin (100 mg/L, Merck), and glacial acetic acid (1 mL/L, Merck). The Petri dishes were cultivated in anaerobic jars with AnaeroGen sachets (Oxoid) at 37 °C for 48 h. *S*. Typhimurium was cultivated aerobically in a 90 mm Petri dish with MacConkey agar (Merck) at 37 °C for 24 h. The BB12 and LT2 CFU were counted from the plates optimally containing 10–100 colonies and 20–200 colonies, respectively.

### 2.5. Ileal Morphometry and Histopathological Evaluation

Ileal morphometry and histopathological score developed for the preterm [41] and the term [42] gnotobiotic piglets were used to assess intestinal morphology. Briefly, the terminal ileum samples were fixed in Carnoy’s fluid for 30 min, dehydrated, and embedded in paraffin. Five μm tissue sections were stained with hematoxylin–eosin and examined under an Olympus BX 40 microscope with a digital camera Olympus Camedia C-2000 (Olympus, Tokyo, Japan), and evaluated as blinded. Averages of 30 evenly spaced radial lamina muscularis propria widths per piglet were determined. Ten measurements for each parameter were taken per piglet to assess ileal villus length and crypt depth. A histopathological score was used: (i) submucosal edema (0–2 score points), (ii) PMNs (polymorphonuclear neutrophils) infiltration into the lamina propria (0–2 score points), (iii) villus atrophy (0–3 score points), (iv) exudate in lumen (0–2 score points), (v) vessels dilatation (0–2 score points), (vi) inflammatory cellularity in lymphatic vessel lumen (0–2 score points), (vii) hyperemia (0–2 score points), (viii) hemorrhage (0–2 score points), (ix) peritonitis (0–1 score points), and (x) erosion of the epithelial layer (0–3 score points). A total score of 0–21 points was obtained calculated.

### 2.6. Intestinal Lavage and Blood Plasma

The intestinal lavages were centrifuged at 2500× *g* for 30 min at 8 °C, and supernatants were filtered through a 0.2 μm nitrocellulose filter (Sartorius, Goettingen, Germany). A citrated blood was centrifuged at 1200× *g* for 10 min at 8 °C. A protease inhibitor cocktail (Roche Diagnostics, Manheim, Germany) was added to both lavages and plasma, and then aliquoted samples were stored at −45 °C until analysis.

### 2.7. Total RNA Isolation and Reverse Transcription

A total RNA was isolated, and cDNA was synthesized as described previously [42]. Briefly, the RNAlater stored cross-sections of the terminal ileum, and transverse colon were homogenized with 2 mm zirconia beads (BioSpec Products, Bartlesville, OK, USA) in 2 mL Eppendorf tubes TissueLyser LT BeadBeater (Qiagen) and total RNA isolated by the RNeasy Plus mini kit (Qiagen). Total RNA (500 ng) was reverse transcribed by QuantiTect reverse transcription kit (Qiagen). The synthesized cDNA was 1/10 diluted with PCR quality water (Life Technologies, Carlsbad, CA, USA) and stored at −25 °C. These PCR templates served for the following real-time PCR.

### 2.8. LNA Probe-Based Real-Time PCR

Two μL of the PCR template and 18 μL of the FastStart Universal Probe Master (Roche Diagnostics) containing 500 nM each of the forward and reverse primers (Generi-Biotech, Hradec Kralove, Czechia), and 100 nM locked nucleic acid (LNA) probe (Universal ProbeLibrary; Roche Diagnostics) were mixed (Table 1). The PCR amplification was performed in duplicates in 45 cycles (95 °C for 15 s and 60 °C for 60 s) ran on an iQ cycler (Bio-Rad, Hercules, CA, USA). Relative mRNA expressions were calculated by the 2^−ΔC^_T_ method [43] and normalized to β-actin and cyclophilin A by GenEx 6 software (MultiD Analyses AB, Gothenburg, Sweden) [42].

### 2.9. Luminex xMAP Technology

Intestinal lavage and plasma levels of IL-8, TNF-α, and IL-10 were measured by a paramagnetic sphere-based xMAP technology (Luminex Corporation, Austin, TX, USA) with a porcine ProcartaPlex kit (Affymetrix, Santa Clara, CA, USA) on the Bio-Plex and evaluated by Bio-Plex Manager 4.01 software (Bio-Rad) as described previously [42].

### 2.10. Statistical Analysis

Differences among the groups in parameters with normal distribution were evaluated with an unpaired *t*-test or one-way analysis of variance (ANOVA) with Tukey’s multiple comparisons post hoc test. Values that did not meet the normal distribution were evaluated with Kruskal–Wallis with Dunn’s multiple comparisons post hoc test. The statistical comparisons were performed at *p* ˂ 0.05 by GraphPad 6 software (GraphPad Software, San Diego, CA, USA) and statistically significant differences depicted in figures by asterisks or a letter system.

## 3. Results

### 3.1. Clinical Signs of Salmonellosis

The GF piglets and piglets associated with BB12 thrived. The piglets infected with *Salmonella* (LT2) showed sleepiness, anorexia, increased body temperature, and profuse non-bloody diarrhea with the onset 10–12 h after the infection with LT2. The piglets previously associated with BB12 (BB12 + LT2) were also diarrheic, but in some cases, their diarrhea was milder.

### 3.2. Colonization of the Intestine with Bifidobacterium animalis Subsp. lactis BB-12 and Its Translocation

**Figure 2:**
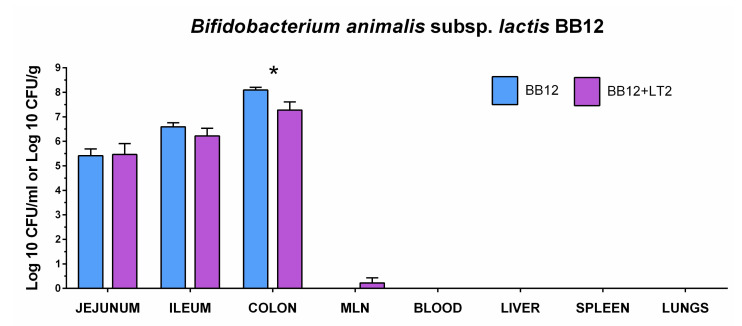
Colony-forming units (CFU) of *B. animalis* subsp. *lactis* BB-12 (BB12) in the gnotobiotic piglets. BB12 CFU was counted in the jejunum, ileum, colon, and blood (all CFU/mL) and mesenteric lymph nodes (MLN), liver, spleen, and lungs (all CFU/g) in the piglets monoassociated with BB12 (BB12; blue column) and the piglets monoassociated with BB12 and one week later infected with *S*. Typhimurium LT2 for 24 h (BB12 + LT2; violet column). Interferences between BB12 and LT2 were evaluated by unpaired *t*-test, and statistical differences were marked with asterisks at *p* < 0.05. Log CFU are depicted as mean + SEM and *n* = 6 piglets for all groups.

We evaluated the colonization of the intestine with *B. animalis* subsp. *lactis* BB-12 (BB12) in one-week-old gnotobiotic piglets (BB12 group) and in their counterparts that were additionally infected with *S*. Typhimurium (BB12 + LT2 group) for 24 h. BB12 successfully colonized the jejunum, ileum, and colon of the GF piglets, and its CFU counts gradually increased through the intestine (Figure 2). The infection with *S*. Typhimurium LT2 (LT2) of one-week-old piglets for 24 h decreased the BB12 counts in the ileum and colon. However, the reduction in the number of BB12 was statistically significant in the colon only. BB12 did not translocate into observed organs or cause bacteremia in the BB12 mono-associated piglets (BB12). The piglets additionally infected with LT2 (BB12 + LT2) demonstrated low-level translocation of BB12 found in MLN.

### 3.3. Colonization of the Intestine with Salmonella Typhimurium LT2 and Its Translocation

**Figure 3:**
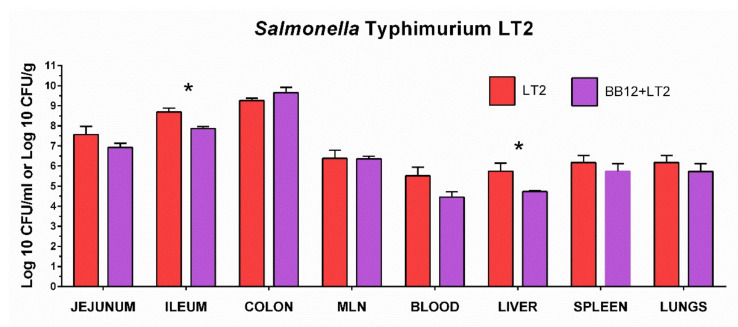
Colony-forming units (CFU) of *S*. Typhimurium LT2 in the gnotobiotic piglets. *S*. Typhimurium LT2 CFU was counted in the jejunum, ileum, colon, and blood (all CFU/mL) and mesenteric lymph nodes (MLN), liver, spleen, and lungs (all CFU/g) in the piglets infected with *S*. Typhimurium LT2 for 24 h (LT2; red column) and the piglets monoassociated with *B. animalis* subsp. *lactis* and one week later infected with *S*. Typhimurium LT2 for 24 h (BB12 + LT2; violet column). Interferences between *B. animalis* subsp. *lactis* BB-12 and *S*. Typhimurium were evaluated by unpaired *t*-test. Statistical differences were marked with asterisks at *p* < 0.05. Log CFU are depicted as mean + SEM and *n* = 6 piglets for all groups.

We studied the influence of *B. animalis* subsp. *lactis* BB-12 (BB12) on multiplication and translocation of *S*. Typhimurium LT2 (LT2) in the gnotobiotic piglets (Figure 3). The LT2 counts gradually increased through the intestine from the jejunum to the colon. The BB12 decreased LT2 counts in all organs, with the exception of the colon and MLN. A statistically significant decrease in the LT2 count in the presence of BB12 was observed in the ileum and liver.

### 3.4. Histopathological Evaluation of the Ileum

It was possible to evaluate villi length, crypt depth, and muscular thickness in the GF and BB12 groups only because the villi structure was destroyed in the LT2 and BB12 + LT2 groups. No statistically significant differences in any of the parameters mentioned above were observed between the GF and BB12 groups.

**Figure 4:**
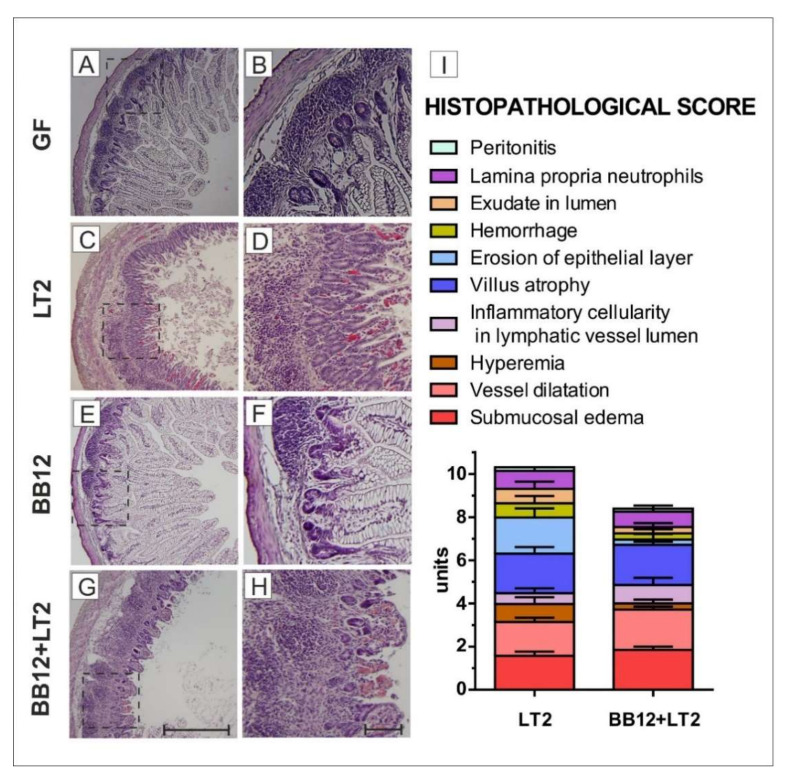
Histopathological evaluation of the ileum in the gnotobiotic piglets: germ-free (GF; **A**,**B**), infected with *S*. Typhimurium LT2 for 24 h (LT2; **C**,**D**), associated with *B. animalis* subsp. *lactis* BB-12 (BB12; **E**,**F**), and associated with BB12 and infected with LT2 for 24 h (BB12 + LT2; **G**,**H**). Bars represent 500 μm (**G**) and 100 μm (**H**), respectively. Histopathological changes affected the LT2 and BB12 + LT2 groups. Histopathological scores from the ileum of six piglets per group are depicted (**I**).

In contrast, the histopathological changes in the ileum only occurred in the *Salmonella*-infected groups (Figure 4). The most obvious difference in the ileum between the non-infected GF (Figure 4A,B) and BB12 (Figure 4E,F) and *Salmonella*-infected LT2 (Figure 4C,D) and LT2 + BB12 (Figure 4G,H) piglets was the disappearance of vacuolated enterocytes in both *Salmonella*-infected groups (Figure 4C,D,G,H). Colonization of piglets with BB12 mildly increased the cellularity in the lamina propria and submucosa (Figure 4E,F) compared to their GF counterparts (Figure 4A,B). Therefore, we did not observe other inflammatory changes in these piglets; both GF and BB12 groups were not included in the calculation of the histopathological score (Figure 4I). The histopathological scoring system showed 10.2 units for the LT2 group and 8.2 units for the BB12 + LT2 piglets (Figure 4I). Hyperemia, hemorrhage, multiple erosion of the ileal epithelium, and the presence of exudate in the lumen characterized the LT2 group (Figure 4C,D). In contrast, the inflammatory cellularity in the lymphatic vessel lumen and rare erosion of the intestinal epithelium characterized the BB12 + LT2 group (Figure 4G,H).

### 3.5. Changes in Claudin-1 and Occludin mRNA Expression in the Ileum and Colon

**Figure 5:**
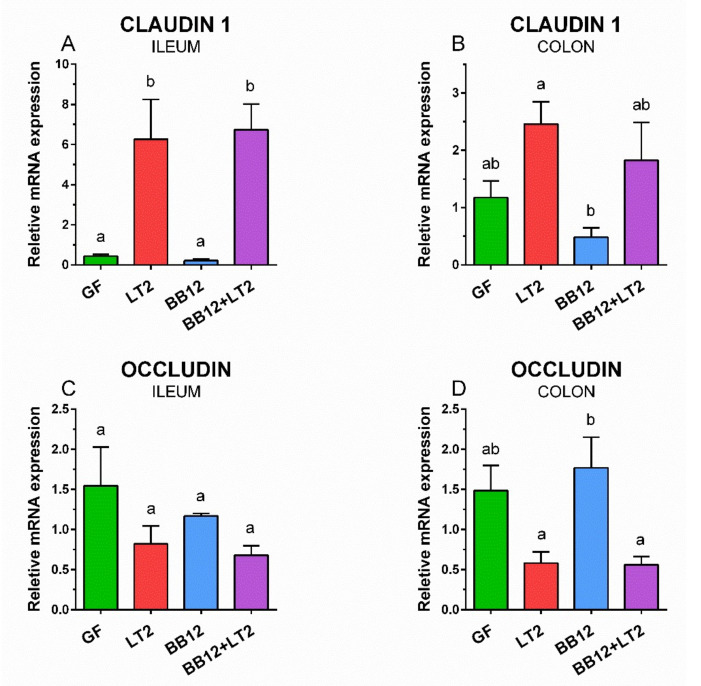
Relative expression (fold change) of claudin-1 (**A**,**B**) and occludin (**C**,**D**) mRNA in the ileum (**A**,**C**) and colon (**B**,**D**) of the gnotobiotic piglets: germ-free (GF), infected with *S*. Typhimurium LT2 for 24 h (LT2), associated with *B. animalis* subsp. *lactis* BB-12 (BB12), and associated with BB12 and infected with LT2 for 24 h (BB12 + LT2). The values are presented as mean + SEM. Statistical differences were calculated by one-way ANOVA with Tukey’s multiple comparison post hoc test, and *p*-values < 0.05 are denoted with different letters above the columns. Six samples in each group were analyzed.

Claudin-1 mRNA expression in the ileum (Figure 5A) was statistically significantly upregulated by the infection with *S*. Typhimurium in the LT2 and BB12 + LT2 groups. A similar trend was observed in the colon, but statistically significant differences were between LT2 and BB12 groups only (Figure 5B). In contrast, *Salmonella* infection seemingly showed the opposite trend for occludin mRNA expression, but without any statistical differences between the infected and non-infected groups in the ileum (Figure 5). This trend of *S*. Typhimurium-induced downregulation of occludin mRNA expression was more obvious in the colon (Figure 5D), and statistically significant differences were found between the BB12 treatment alone and both LT2-infected (LT2 and BB12 + LT2) groups.

### 3.6. Intestinal Inflammatory Cytokine Concentrations

**Figure 6:**
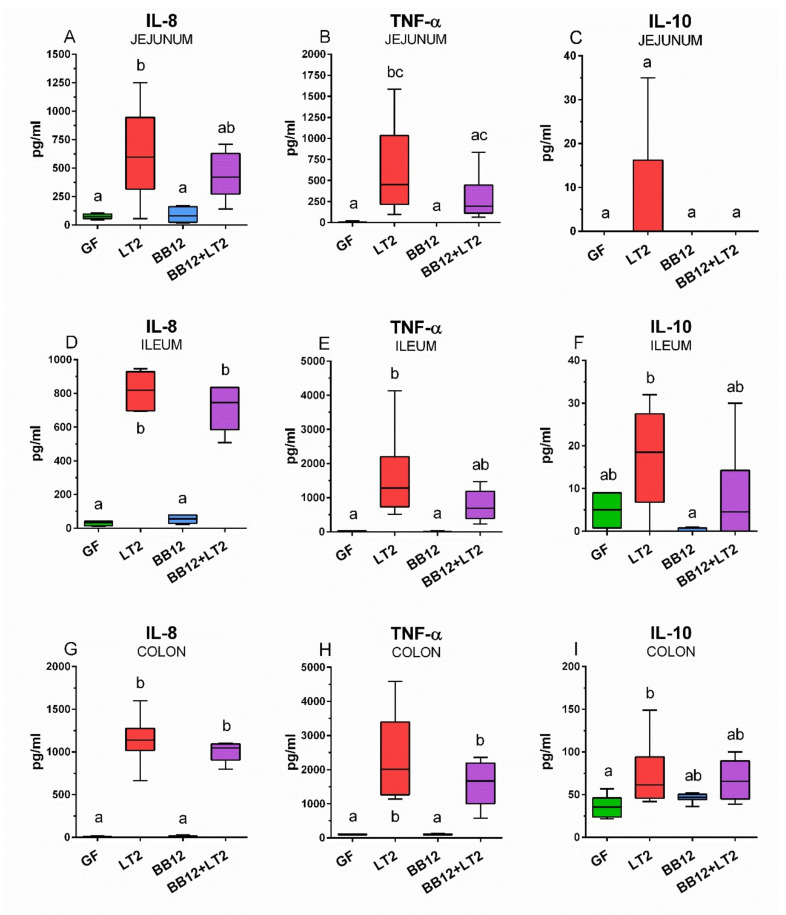
Intestinal levels of the inflammatory cytokines IL-8 (**A**,**D**,**G**), TNF-α (**B**,**E**,**H**) and IL-10 (**C**,**F**,**I**) in the jejunum (**A**–**C**), ileum (**D**–**F**), and colon (**G**–**I**) of the gnotobiotic piglets: germ-free (GF), infected with *S*. Typhimurium LT2 for 24 h (LT2), associated with *B. animalis* subsp. *lactis* BB-12 (BB12), and associated with BB12 and infected with LT2 for 24 h (BB12 + LT2). The values are presented as boxes indicating the lower and upper quartiles, the central line is the median, and the ends of the whiskers depict the minimal and maximal values. Statistical differences were calculated by the Kruskal–Wallis test with Dunn’s multiple comparison post hoc test, and *p* < 0.05 are denoted with different letters above the columns. Six samples in each group were analyzed.

The association of the GF piglets with the BB12 alone did not induce inflammatory cytokines compared to GF (Figure 6). Infection with *S*. Typhimurium alone (LT2 group) significantly induced IL-8 levels in all observed parts of the intestine (Figure 6A,D,G). Prior association with BB12 (BB12 + LT2 group) ameliorated the *Salmonella* induction of IL-8 in the jejunum, but not in the ileum and colon, where the IL-8 levels were significantly higher as in the LT2 group. Infection with *S*. Typhimurium alone (LT2 group) significantly induced TNF-α in all parts of the intestine (Figure 6B,E,H) and in the colon of the BB12 + LT2 piglet group (Figure 6H). The levels were also increased in the jejunum and ileum (Figure 6B,F) but were not statistically significantly different from GF. At the same time, these levels did not show statistically significant differences from the levels of the LT2 group in all observed parts of the intestine (Figure 6B,E,H). IL-l0 was not significantly increased in the jejunum (Figure 6C). In the ileum and colon, IL-10 concentration was significantly increased in the piglets infected with *S*. Typhimurium alone (LT2 group), but not in the group previously associated with BB12 (Figure 6F,I). However, this increase did not show statistically significant differences from the GF or BB12 groups.

### 3.7. Plasma Inflammatory Cytokine Concentrations

**Figure 7:**
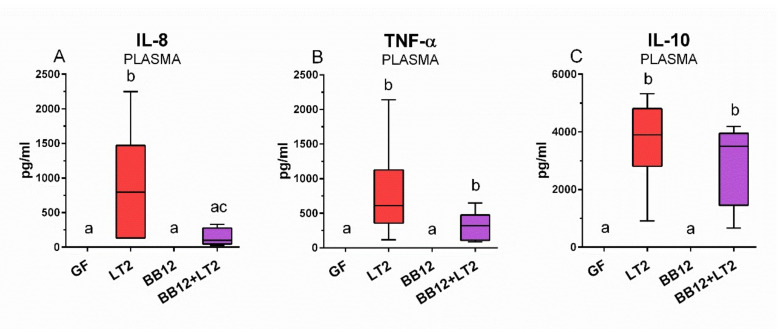
Plasmatic levels of the inflammatory cytokines IL-8 (**A**), TNF-α (**B**) and IL-10 (**C**) in the gnotobiotic piglets: germ-free (GF), infected with *S*. Typhimurium LT2 for 24 h (LT2), associated with *B. animalis* subsp. *lactis* BB12 (BB12), and associated with BB12 and infected with LT2 for 24 h (BB12 + LT2). The values are presented as boxes indicating the lower and upper quartiles, the central line is the median, and the ends of the whiskers depict the minimal and maximal values. Statistical differences were calculated by the Kruskal–Wallis test with Dunn’s multiple comparison post hoc test, and *p* < 0.05 are denoted with different letters above the columns. Six samples in each group were analyzed.

Plasma IL-8, TNF-α, and IL-10 concentrations were statistically significantly increased in piglets infected with *S*. Typhimurium alone (LT2 group) (Figure 7). *S*. Typhimurium also induced levels of all cytokines in the piglets previously associated with *B*. *animalis* subsp. *lactis* BB-12, and this increase was statistically significant for TNF-α and IL-10 (Figure 7B,C).

## 4. Discussion

Preterm infants are born prior to 37 weeks of gestation, which results in low birth weight, underdeveloped organ function, including immune development, which makes them susceptible to many life-threatening comorbidities. Reduced digestive function, intestinal motility, and underdeveloped intestinal barrier can result in prolonging exposition to various toxins and increased bacterial adherence to the intestinal lining, which can predispose preterm infants to excessive fermentation and bacterial overgrowth with increased translocation [44,45]. The preterm piglet model is used for the simulation of necrotizing enterocolitis (NEC) in infants [8]. Based on organ development, it is estimated that 90% gestation in the pig corresponds to 75% gestation (30–32 weeks) in the human [46]. Preterm infants may also have delayed exposure to colostrum from their own mother or may never receive colostrum if fed donor human milk. In the GF piglet, deprivation of colostrum and the absence of the conventional indigenous microbiota deepen the immunocompromised of the piglets and their breeding in microbiologically controlled conditions of gnotobiotic isolators allow studies of microbial interferences among defined microbiota and its impact on the health of the host [21,41] and also recapitulate the preterm infant physiological state and environment.

*Bifidobacterium* spp. are members of human and animal microbiota located mainly in the gastrointestinal tract [47]. As strict anaerobes, they need suitable conditions created by pioneer facultative anaerobic bacteria [1]. Interestingly, the bifidobacteria can be cultivated from the feces of vaginally born children from the first day, and their number in feces gradually increased for the whole three-year observed period [48]. Various bifidobacterial species are also represented in the pig microbiome [49,50]. Probiotic *Lactobacillus rhamnosus* GG (LGG) and *B. animalis* subsp. *lactis* BB-12 (BB12) have been previously demonstrated to successfully colonize the GF piglet intestine [51]. Other experiments proved the possibility to monoassociate the GF piglets with pig indigenous *B. choerinum* [52] and *B. boum* [40] without the support of any aerotolerant oxygen-consuming bacteria.

The most frequently used probiotics belong to genera *Bifidobacterium*, *Escherichia*, *Lactobacillus*, and *Streptococcus* [53]. In preterm infants, only a minority of the studied individual strains or strain combinations have effectively reduced mortality and morbidity [54]. Both the evidence for xenotransplantation of the microbiota to the GF piglets [15,16] and monoassociation with indigenous pig bifidobacteria [40,52] suggested the possibility to monoassociate the GF piglet intestine with probiotic BB12. This *Bifidobacterium* belongs to a diverse group of *B. animalis* subsp. *lactis* isolated from different host species [55] and is one of the most frequently used probiotic bacteria in humans [56]. In our former experiments, we verified the safety of *L. rhamnosus* GG and *E. coli* Nissle 1917 for the gnotobiotic piglets and compared their protective effect against infection with *S*. Typhimurium [41,42]. Probiotics per se may rarely be responsible for various side effects as systemic infections, deleterious metabolic activities, excessive immune stimulation, and antibiotic resistance gene transfer [53]. Thus, the presented work completes verification of the safety and efficacy of a triumvirate of well-known and broadly used probiotics *Lactobacillus rhamnosus* GG, *E. coli* Nissle 1917, and *B. animalis* subsp. *lactis* BB-12 [56,57] in the gnotobiotic piglet. In this case, we used commonly available probiotic BB12 that was isolated from a commercial preparation. We sought to verify three hypotheses: (i) BB12 can colonize the preterm GF pig intestine without the support of any oxygen-consuming bacteria, (ii) BB12 is not harmful to the preterm GF piglets, and (iii) BB12 protects the preterm gnotobiotic piglets against infection with *S*. Typhimurium LT2 strain. *S*. Typhimurium commonly causes self-limiting, non-bloody diarrhea in otherwise healthy individuals. However, it can cause life-threatening bloodstream infections and translocation of the organism to other tissues of immunocompromised individuals [36]. The LT2 strain used herein is slightly virulent only for conventional piglets [58] but lethal for GF piglets [59].

BB12 showed its ability to colonize the preterm GF piglet intestine without the presence of any other commensal bacteria, as previously shown for pig indigenous *B. choerinum* and *B. boum* in term piglets [40,52]. The counts of BB12 in the presence of LT2 were comparable in the jejunum and ileum but were lower in the colon in comparison with the BB12 monoassociated piglets. The colon is the prominent region of the gut for the activity of bifidobacteria against enteric pathogens, and their main means of combatting infection is via the production of acetic acid [60]. Paradoxically, *Salmonella* is a sophisticated enteric pathogen that can use acetate to increase the expression of its invasive genes and, thus, manipulates both the microbiota and the host for its profit [61,62]. The observed decrease of the colonic BB12 counts in the presence of *Salmonella* is in concordance with the decreased bifidobacteria counts count in the colon of the term gnotobiotic piglets monoassociated with *B. choerinum* [52] as well as the mucolytic or non-mucolytic *B. boum* [40]. The present experiments confirmed this phenomenon in the preterm colon. A possible explanation can be the fact that *Salmonella* can induce the host immune cells to produce reactive nitrogen and oxygen species that oxidize the colonic environment and creates unsuitable conditions for the multiplication and spread of the strictly anaerobic bifidobacteria into the host’s intestine [23,61].

We also considered the concern that probiotics can translocate in critically ill preterm neonates with compromised intestinal barrier integrity [63]. Such infants are at higher risk of probiotic sepsis [64]. However, BB12 did not translocate to MLN, liver, spleen, and lungs and did not cause bacteremia in the BB12 monoassociated intestine. In the BB12 monoassociated intestine and later infected with LT2, BB12 rarely translocated to MLN, probably via a disrupted intestinal barrier due to the *Salmonella* infection. However, BB12 was trapped in MLN and did not spread to other organs, which documents the safety of BB12 for the immunocompromised host. We observed that LT2 growth was diminished by BB12 in most cases, except for the colon, but this reduction was significant in the ileum and liver only. This finding is interesting, as it is believed that the main site of bifidobacteria action is in the colon [60]. In contrast, the main site of translocation of the non-typhoidal *S*. Typhimurium is the ileum [65]. Both the ileum and colon are the most affected parts of the intestine in salmonellosis [66]. Concordantly, the ileum and colon are the same parts of the intestine affected in NEC, the illness jeopardizing the health of preterm infants, and that is probably related to the dysbiotic microbiome [67]. The use of probiotics can reduce the incidence of NEC in preterm infants [68].

The intestinal barrier separates the bacteria-rich intestinal lumen and the sterile organism [63]. *Salmonella* infection disrupts this barrier [69]. We evaluated the changes in the ileum by the histopathological score. Colonization with BB12 prior to LTS infection mildly improved this score and showed a similar protective effect as LGG [41]. The increased sensitivity of the preterm piglet to LT2 was documented by the presence of hemorrhage, which was absent in their term counterparts [42]. The disruption of the intestinal barrier facilitates *Salmonella* invasion of epithelial cells that triggers the recruitment of neutrophils to the inflamed site [70,71]. Neutrophils are the main effector cells in the infection with non-typhoidal *Salmonella* [69]. They are recruited to the inflammatory site by various chemoattractants. Probably the most known is the chemotactic cytokine (chemokine) IL-8 participating as in the attraction and activation of neutrophils [72]. Gram-negative bacteria with rough lipopolysaccharide (LPS) chemotype with incomplete LPS chain are effective inducers of IL-8 and provide a protective effect against infection with virulent *Salmonella* in the gnotobiotic piglets [71]. Thus, increased local levels of IL-8 are important to combat infection [73,74].

Epithelial cells form the host–microbiota interface. They are joined at their lateral surfaces by tight junction proteins, e.g., claudins and occludin, which make this interface semipermeable [63]. Disruption of this interface results in increased bacterial translocation and penetration of toxins into the host organism [75]. Claudins are groups of about 30 known proteins in humans and can be roughly divided into barrier-forming, e.g., claudins 1 and 4, and pore-forming, e.g., claudins 2 and 10 [76]. A decreased expression of the claudin-1 protein was found in the ileum of conventional piglets infected with *Salmonella* Infantis [76]. The upregulation of claudin-1 mRNA expression by the infection with LT2 probably prevented the loss of electrolytes that occurs in diarrhea [75]. Concomitant with claudin-1 upregulation in both the ileum and colon, occludin mRNA was not influenced in the ileum but significantly downregulated in the colon. It is in concordance with non-influenced occludin protein expression in the ileum of the conventional piglets infected with *S*. Infantis [77]. Occludin relates to the transmission of macromolecules [78] and cell migration [79], but its physiological role is less obvious compare to claudins. In physiological conditions, occludin is highly phosphorylated [80]. Infection and infection-induced inflammatory cytokines are responsible for its partial dephosphorylation that influences its participation in the normal function of the intestinal barrier [72]. Similar upregulation of claudin-1 and downregulation of occludin mRNA were observed in the term gnotobiotic piglets [40,42], and proportional changes were related to the virulence of the used *S*. Typhimurium according to the completeness of LPS [71]. Both LT2-infected piglet groups in our experiments suffered from diarrhea, and the previous monoassociation with B12 did not significantly alleviate it.

Cytokines are communication molecules that normally participate in the physiological processes of the host’s organism. Their production can be dramatically increased in infection and tissue damage, and their excessive production provokes detrimental multiple organ dysfunction that can result in death [81]. Thus, inflammatory cytokine levels are used as sepsis markers [82]. The immature GIT has underdeveloped properties and functions, e.g., lower gastric acidity in the stomach, production of bile acids, peristalsis, luminal mucus secretion by goblet cells, the intestinal epithelial cell layer, cell populations in the underlying lamina propria, production of IgA, and microbiome composition that facilitate translocation of pathogens and its products [83,84]. The immature intestine also shows an inappropriately regulated cytokine network that may result in different basal cytokine levels in the unstimulated intestine [21] or inappropriate response to various inflammatory stimuli, including infections [83,84]. Higher levels of the inflammatory cytokines were found in the preterm GF piglets, while in the term piglets, levels were lower or nondetectable [21]. We measured local and systemic levels of the chemokine IL-8 [85], the proinflammatory mediator of a broad range of biological activities, TNF-α [86], and anti-inflammatory mediator IL-10, which prevents over-exuberant response to pathogens [87]. These cytokines are recommended as early markers of sepsis in infants [82] and are also useful in gnotobiotic piglets [88]. Moreover, an increased ratio of plasma IL-10-to-TNF-α prognoses poor clinical outcomes in preterm infants, children, and adults [89,90,91]. IL-8 was increased in all parts of the intestine by *Salmonella* infection. Previous colonization with BB12 did not significantly reduce this augmentation. It is in concordance with the mild effect of LGG in preterm [41] and term gnotobiotic piglets associated with indigenous commensal bifidobacteria [40,52] or lactobacilli [42]. These findings are in contrast with the outcome of association with the probiotic *E. coli* Nissle 1917 prior to *S*. Typhimurium infection [42,52]. The influence of the infection with *S*. Typhimurium and the absence of significant downregulation was observed in TNF-α. In the case of IL-10, the levels of IL-10 were very low in the jejunum and ileum of term piglets but moderate in the colon. The levels of cytokines in the blood mirrored the immune response in the host organism. The plasma levels of all observed cytokines were increased in *Salmonella*-infected piglets, and reduction by the presence of BB12 did not significantly contribute to their downregulation that was described in *E. coli* Nissle 1917 [42,52]. The appearance of IL-10 in the plasma of in gnotobiotic piglets is associated with a poor prognosis for survival [92] that is in agreement with findings in humans [90,91].

We can summarize that infection with *S.* Typhimurium LT2-induced sepsis in the preterm gnotobiotic piglets. The previous association with probiotic *B. animalis* subsp. *lactis* BB-12 mildly alleviated the consequences of the infection.

## 5. Conclusions

A balanced GIT microbiota is a prerequisite for host health. This balance is relatively fragile, and it can be shifted to dysbiosis in several ways, such as infection and use of antibiotics. Cesarean section-born preterm infants have a vulnerable intestinal niche that should be appropriately colonized to support optimal development and avoid life-threatening complications that are associated with dysbiosis. A defined multispecies microbiota is likely the best way to fulfill the infant’s physiological requirements without endangering the immunocompromised preterm host. The experimentally infected preterm gnotobiotic piglet model can serve for experiments that are unethical in humans and that can provide important knowledge for translational research. We have shown that the germ-free colostrum-deprived preterm piglets represent a valuable model for studying the effects of the pathogen in the condition of an immunocompromised host. The understanding of the interaction of intestinal infection and probiotic strains will be necessary to develop novel approaches to the targeted modulation of microbiota. Future experiments are needed to identify the best probiotic that will exert effective protection against pathogens.

## Figures and Tables

**Table 1:** Locked nucleic acid (LNA) probe-based real-time PCR systems.

Gene	5′-Forward Primer-3′	5′-Reverse Primer-3′	#LNA Probe
BACT ^1^	TCCCTGGAGAAGAGCTACGA	AAGAGCGCCTCTGGACAC	9
CYPA ^2^	CCTGAAGCATACGGGTCCT	AAAGACCACATGTTTGCCATC	48
CLD-1 ^3^	CACCACTTTGCAAGCAACC	TGGCCACAAAGATGGCTATT	3
OCLN ^4^	AAAGAGCTCTCTCGACTGGATAAA	AGCAGCAGCCATGTACTCTTC	42

^1^ β-actin, ^2^ cyclophylin A, ^3^ claudin-1, ^4^ occludin.

## Data Availability

Data are available on request from the corresponding author.
